# Does neoadjuvant chemotherapy increase breast conservation in operable breast cancer: an Egyptian experience

**DOI:** 10.3332/ecancer.2008.104

**Published:** 2009-04-09

**Authors:** N Abdel-Bary, AF El-Kased, HAZ Aiad

**Affiliations:** 1 Department of Clinical Oncology, Faculty of Medicine, Menofia University, Egypt; 2 Department of Surgery, Faculty of Medicine, Menofia University, Egypt; 3 Department of Pathology, Faculty of Medicine, Menofia University, Egypt

## Abstract

**Introduction:**

The role of adjuvant chemotherapy in breast cancer is well established, as are its indications. Likewise, the role of neoadjuvant chemotherapy in locally advanced breast cancer is well established. The use of neoadjuvant chemotherapy in operable breast cancer has only recently become of interest to researchers.

**Patients and methods:**

This study included 34 cases of operable breast cancer that were given four cycles of neoadjuvant chemotherapy in the form of FEC100 then subjected to surgery. The surgery done was either breast conserving surgery or modified radical mastectomy. All patients completed the treatment regimen and no patients were excluded from the study. All surgical specimens were studied pathologically for chemotherapy effect.

**Results:**

An overall objective response was observed in 70.6% of the patients. Seven patients (20.6%) experienced a clinical complete response (cCR), 17 patients (50.0%) had partial response, nine patients (26.5%) had no change of their disease and only one patient had disease progression. Of the seven patients who had a cCR, only four patients (11.8%) had pathologic complete response (pCR), while pCR for the whole group was 14.7%(5/34). Tumour size of more than 2 cm was observed in 28 patients (82.4%) at time of presentation, while tumour size of 2 cm or less was seen in six patients (17.6%) only. After completion of the course of chemotherapy, 23 patients (67.6%) were observed to have tumours of 2 cm or less that allowed for less extensive resections. Twenty-three patients underwent breast conservative surgery (67.6%) while modified radical mastectomy was performed in 11 patients (32.4%).

**Conclusion:**

The use of neoadjuvant chemotherapy in operable breast cancer in this study was associated with tumour and axillary downstaging, which increased the proportion of cases undergoing breast conservation, with acceptable side effects and reasonable cost. During the limited follow-up time of this study no loco regional recurrences were recorded and one distant treatment failure was recorded. Its impact if any on overall or disease-free survival was not addressed in this study. Larger multi-centre randomized studies with a long follow-up are needed to compare the overall and disease-free survival benefit of this treatment modality, especially in different subtypes stratified by pathological response.

## Introduction

Trials that studied the role of adjuvant chemotherapy in the management of primary operable breast cancer conducted during the 1970s and 1980s showed significant improvements in progression-free and overall survival [1]. Conventionally, adjuvant systemic therapy is administered after local treatment in early breast cancer [2]. The role of neoadjuvant chemotherapy and endocrine therapy in locally advanced breast cancer has also become well established [3]. However, since the introduction of conservative treatment modalities, there has been considerable interest in the efficacy of preoperative chemotherapy to decrease tumour size. One of the potential benefits of preoperative chemotherapy is the more frequent usage of breast-conserving treatment modalities [4]. Moreover, it has been hypothesized that preoperative chemotherapy may have a more powerful effect on survival compared with post-operative chemotherapy. It was found that animal models treated with chemotherapy or tamoxifen prior to surgical resection have improved survival, presumed as a result of a reduction in dissemination of cancer cells following surgery [5–7]. Successful early treatment with systemic therapy is consistent with the Goldie-Coldman hypothesis, whereby metastases are being treated prior to the emergence of chemoresistant mutant clones [8].

Although the merits of preoperative chemotherapy in the treatment of locally advanced breast cancer are well established, the feasibility of preoperative chemotherapy in early breast cancer is still a matter of discussion.

The European Organization for Research and Treatment of Cancer (EORTC) Breast Cancer Cooperative Group started a randomized trial in 1991 (EORTC trial 10902) to investigate the value of preoperative chemotherapy in early breast cancer and concluded that there is no difference in terms of overall survival or relapse-free survival between pre- and post-operative chemotherapy in early breast cancer. The data also concluded that, in such setting, preoperative chemotherapy allows better breast conservation. Survival data from the much larger NSABP B18 trial, with 1523 patients randomized to four cycles of adriamycin plus cyclophosphamide before or after surgery, have recently been reported with no significant differences in overall survival or relapse-free survival.

The study presented here assesses the clinical as well as pathological response of breast cancer to a preoperative FEC-100 regimen (fluorouracil 500 mg/m^2^, epirubicin 100 mg/m^2^, cyclophosphamide 500 mg/m^2^ administered intravenously), and its significance in reducing the need for mastectomy and increasing the potential for more conservative breast surgery at the Menofia Oncology Hospital, Egypt.

## Patients and methods

### Patient characteristics

Thirty-four women with breast cancer attending the Department of Surgical Oncology at the Menofia Oncology Hospital were enrolled into this study from June 2004 to November 2006. Patients had primary operable breast cancer (T1 to T3, N0 to 1 and M0). Breast cancer was diagnosed by core-needle biopsy in all cases even if the patient had been previously diagnosed by fine-needle aspiration. A complete history was taken for all patients at the first presentation and a complete physical examination (including careful assessment of breast as well as axillary lymph nodes), routine laboratory tests, including CBC, liver and renal function tests, alkaline phosphatase, radiological tests, including a chest x-ray, pelvic-abdominal ultrasound, bilateral mammography with confirmatory breast ultrasound and complete echocardiography with assessment of the ejection fraction were carried out at this point and after the completion of the course of chemotherapy to assess treatment response. CBC was performed prior to each cycle of chemotherapy.

Exclusion criteria consisted of the following:

- more than 70 years old;
- lumpectomy or axillary nodal biopsy;
- bilateral breast cancer;
- previous treatment for breast cancer;
- presence of distant metastases;
- pregnancy or lactation at the time of diagnosis;
- previous or current other malignancies;
- World Health Organization performance status more than 2;
- active cardiac disease (including EF below 55%);
- severe haematological, renal or hepatic abnormalities.

All patients gave informed consent before entering the trial.

### Treatment

Treatment consisted of four cycles of the FEC-100 regimen followed by surgery. Decision on the type of surgery (whether breast conserving surgery or mastectomy) was taken on the final assessment of the disease after the fourth cycle.

The chemotherapy regimen consisted of four cycles of preoperative fluorouracil 500 mg/m^2^, epirubicin 100 mg/m^2^ and cyclophosphamide 500 mg/m^2^ (FEC-100) administered intravenously, at three-week interval. Administration of FEC was delayed for a maximum of two weeks in the case of haematological, hepatic, renal or gastrointestinal toxicity. Dose modifications followed the guidelines stipulated by the EORTC Breast Cancer Cooperative Group [10].

Surgery was planned to be performed within 4–6 weeks of the fourth course of chemotherapy.

### Tumour response

Clinical tumour size and nodal status were estimated before the start of chemotherapy as well as at the time of surgery by both palpation and mammography. The product of the two greatest perpendicular diameters was used to compare tumour size before and after chemotherapy, as defined by the International Union Against Cancer criteria [11]. A complete clinical response (cCR) was considered a complete disappearance of all clinically detectable malignant disease by palpation as well as mammography. The clinical response to chemotherapy was assessed before each cycle and at the time of surgery. If the tumour had become undetectable before completion of the four cycles of preoperative chemotherapy, chemotherapy was continued as outlined in the protocol. Clinical partial response was defined as 50% decrease in total tumour size after four cycles of chemotherapy at the time of surgery. An increase of 25% in tumour size after a minimum of two courses of preoperative chemotherapy was considered to be progressive disease (PD). In patients with clinically negative nodes at presentation, the development of palpable nodes during the administration of chemotherapy was considered evidence of PD. After a diagnosis of PD, patients immediately underwent surgery before completing the preoperative chemotherapy schedule. If patients did not meet one of the above-mentioned criteria after four cycles of chemotherapy, they were classified as having stable disease. Tumour specimens were examined pathologically to assess the response and compare clinical to pathologic response. If no signs of residual malignant cells at the primary site and axillary lymph nodes were seen with histological examination, this was scored as a pathologic complete response (pCR).

### Pathology

The surgical specimens were resected and oriented according to a defined surgical protocol described in the UK Guidelines [12]. For the therapeutic wide local excisions, once in the laboratory, the entire surface of the specimen was stained so that the margins of excision could be easily determined. The system followed to record the clinical response to neoadjuvant therapy was that of the International Union Against Cancer [11]. The histopathological response to neoadjuvant therapy was assessed according to criteria suggested by Smith *et al* [13]. These authors suggest that the features of the primary tumour should be scored from G1 to G5:

1. no reduction in overall numbers of tumour cells compared with pre-treatment core biopsy;
2. mild loss of tumour cells, but overall cellularity remaining high;
3. up to 90% reduction in tumour cells;
4. marked disappearance with only small clusters remaining;
5. no invasive tumour, *in situ* carcinoma or stromal reaction remaining.

Similarly, the lymph nodes are categorized as follows:

- true negative, no metastasis and no alterations;
- metastasis with no histological alterations;
- metastasis with alterations;
- no metastasis with alterations.

For classification into these categories, it was essential that an invasive tumour was identified. This necessitated the detection of abnormal fibroelastic breast stroma that was devoid of normal lobular units and contained foamy macrophages, a moderate numbers of fibroblasts and other mononuclear inflammatory cells. Grade 5 response was deemed to represent a pCR of the primary cancer.

Oestrogen and progesterone receptor status were ascertained in all specimens by immunohistochemical staining. In brief 5-μm-thick sections of paraffin-embedded blocks were deparaffinized, dehydrated and then placed in citrate buffered saline (pH 6.0) and boiled for 20 minutes. Endogenous peroxidase activity was blocked by incubation with 6% H_2_O_2_ in methanol. The primary antibodies used were mouse monoclonal anti-human oestrogen receptor alpha, clone 1D5 (ready to use), mouse monoclonal anti-human progesterone receptor, clone PgR 636 (ready to use) and concentrated rabbit polyclonal anti-human C-erb-B-2 oncoprotein (Her2/neu) (1:50), Hercept test, Code No. A0485 (Dako, Copenhagen, Denmark). The primary antibodies were incubated overnight. Immunoreactivity was visualized using Envision + (Dako cytomation, Glostrup, Denmark) with DAB chromogen as substrate and Mayer’s haematoxylin as counterstain. Previous positive breast carcinoma for ER, PR and C-erb-B-2 were used as positive control for ER, PR and C-erb-B-2, respectively. Negative controls were prepared by substituting the primary antibodies with saline.

Evaluation of immunostaining: nuclear staining was a prerequisite for assigning ER and PR positivity; both the number of tumour cell nuclei and the intensity of the reaction were evaluated. For C-erb-B-2, complete membranous staining in more than 10% was required for positivity.

## Results

Thirty-four patients were enrolled in this study, patient characteristics are shown in Table 1.

All patients received the planned chemotherapy in time except for two patients who had severe neutopenia requiring colony stimulating factors and treatment delay (one patient delayed for one week and the other for ten days).

Chemotherapy was well tolerated with acceptable toxicities.

Mild to moderate toxicity that is temporary and improves on simple and ordinary treatment is assigned as transient, while severe toxicity requiring specific aggressive treatment or admission is assigned as severe. Toxicity resulting from chemotherapy is shown in Table 2.

Tumour size of more than 2 cm was observed in 28 patients (82.4%) at time of presentation, while only six patients (17.6%) had a tumour size of 2 cm or less. After completion of the course of chemotherapy 23 patients (67.6%) had tumours of 2 cm or less allowing less extensive resections.

## Surgery

All patients underwent surgery within 35 days of the fourth cycle (average 19 days) in the form of conservative breast surgery in 23 (67.6%) or modified radical mastectomy in 11 (32.4%) patients.

Decision to perform breast conservative surgery (BCS) or modified radical mastectomy was based on the tumour size, breast tumour ratio and tumour position within the breast as shown in Table 3. Modified radical mastectomy was not followed by immediate reconstruction. Perioperative antibiotics were given to all patients and suction drains were used. Routine second day discharge from hospital with the drains was done for all patients. One lumpectomy patient was readmitted five days later for wider excision due to involved margins in the final report in spite of negative margins by frozen section.

In patients undergoing BCS wide excision, staining of frozen sections was performed to control the margins. A problem was met in patients with cCR where no palpable tumour was present. In these patients, a quadrantectomy was done where the index quadrant of the tumour was excised. All cases undergoing BCS underwent full axillary dissection through a separate transverse lower axillary crease incision.

Patients with a partial response lumpectomy was usually possible guided by the palpable lump although in three cases the breast mass was non-palpable and excision was performed after preoperative wire localization. Perioperative antibiotics were used for all patients, routine axillary suction drains were used while lumpectomy cavity drains were only used selectively. No mortalities were observed and only minor morbidity in the form of wound infection (two cases cleared spontaneously on treatment) and seroma (two cases in the mastectomy group required several aspirations and two cases of axillary seroma in the BCS group required aspiration and drain reinsertion in one case). The cosmetic results were evaluated as regard the appearance of the scars, the position and size of the areola-nipple complex and the size of the breast in comparison with the other breast as very good (+++), moderate (++) or fair (+).

In the BCS group, patient satisfaction with final cosmetic appearance was very satisfactory. The quadrantectomy patients were less satisfied with the final outcome than those undergoing lumpectomy.

### Tumour response

An overall objective response was observed in 70.6% of the patients. Seven patients (20.6%) experienced a cCR, 17 patients (50.0%) a partial response, nine patients (26.5%) had stable disease and one patient had disease progression (see Table 4).

Pathologic complete response (pCR = grade 5) was seen in five (14.7%) patients and a pPR (grade 1–4) in 29 patients (Table 5). There was no axillary lymph node involvement after chemotherapy in 26 patients (76.5%), of them true negative (category A) represented (58.8%) and signs of tumour regression (nodal fibrosis, mucin pools, aggregates of foamy histiocytes, myxoid or mucinous areas) were seen in five patients (category D). No response (category B) was observed in five patients (14.7%) and nodes with metastasis associated with histological alterations were observed in three patients (8.8%).

A tumour size of more than 2 cm was observed in 28 patients (82.4%) at the time of presentation, while a tumour size of 2 cm or less was seen in only five patients (17.6%). After completion of the course of chemotherapy, 23 patients (67.6%) were observed to have tumours of 2 cm or less so allowing for less extensive resections. Pathologic examination of axillary nodes revealed negative nodes in 26 patients and positive nodes in eight patients. Patients with positive nodes showed signs of regression while signs of complete tumour necrosis (burnt-out tumour) were seen in five patients with negative nodes.

### Further management

Patients who had a good biological (pathological) response received two more cycles of the same regimen (FEC100) to a total of six cycles. Those who had a poor biological response were shifted to a taxane-containing regimen (docitaxel and cisplatin). All patients who underwent BCS received radiotherapy to the residual breast and chest wall as well as peripheral lymphatics (when indicated). Doses of radiotherapy to the residual breast and chest wall were 5000cGy in 25 fractions over five weeks followed by a booster dose of 1500 to 2000 cGy in 7–10 fractions over 10–15 days. Hormonal therapy was given to hormone responsive tumours. Follow-up of patients ranged from 6 to 28 months. No local recurrences were seen in any of our patients during this limited follow-up. One case developed distant metastasis (lung) 14 months after completion of therapy.

## Discussion

Neoadjuvant and adjuvant chemotherapy for breast cancer have been compared in several previous clinical trials, but these trials did not definitively show that one approach was better than the other. The rationale for the use of neoadjuvant chemotherapy is: (1) the ability to reduce the extent of surgical procedures so, allowing for better cosmoses, (2) the ability to assess clinical as well as biological response of the given treatment, (3) eradication of micro-metastases, (4) to allow precise pathologic assessment of tumour response and (5) an opportunity for a scientific study of serial pre- and post-treatment tumour biopsy samples [14].

The use of preoperative or primary chemotherapy was introduced approximately three decades ago in locally advanced breast cancer. Since then, its role in the management of locally advanced breast cancer has been firmly established. However, the advantages are not clear in early breast cancer [15]. Despite the fact that preoperative chemotherapy may permit more breast-conserving treatment modalities, there may be problems, for instance in achieving adequate loco-regional control as a result of the difficulty of assessing tumour margins after the administration of preoperative chemotherapy [16].

Objective clinical response to neoadjuvant chemotherapy is reported to be between 65% and 91%. In our series, clinical response was seen in 70.6% of our cases, which is in accordance with other published reports [17,18]. Reported cCR rates are much lower ranging from 10% to 30% in most series, although some authors have reported a much lower rate. In our patients, cCR was seen in 20.6% of the cases, which is in accordance with most published reports [14]. Pathological complete responses are usually in the 9–15% range although there are some reports citing up to 30% pathological response rates. The reason for this discrepancy is usually because these authors include minimal disease (or only microscopic foci of disease) in their figures for cPR [19–21]. We were able to detect a cPR in 14.7% of our cases, which is also in agreement with most of the published data.

Axillary downstaging is also reported to be a good prognostic factor. A primary tumour response is usually associated with a good axillary response. In our patients, 41.2% had palpable axillary nodes on presentation. After the end of chemotherapy, only 26.5% had palpable nodes.

Although the benefits of preoperative chemotherapy in early breast cancer patients are less clear compared with the locally advanced breast cancer patients, the potential to enhance breast-conserving therapy makes it an attractive treatment modality. Several authors have firmly established the reduced need for mastectomy with neoadjuvant chemotherapy or neoadjuvant endocrine therapy [19,22,23]. In our experience, mastectomy was performed in only 32.4% of cases compared to a mastectomy rate of almost 80% observed at the Menofia University Cancer Center. This is in accordance with the finding of other authors who showed a significant reduction in the need for mastectomy; Schwartz *et al* [19] observed a 62% rate of breast conservation while Makaris *et al* reported an 89% rate of breast conservation with neoadjuvant chemotherapy compared to 78% when neoadjuvant chemotherapy was not used [19,23].

Complications after surgery were only minimal with no significant differences between patients who underwent a mastectomy and those who had BCS. The two major problems encountered by us were: (1) the difficulty in assessing margins by frozen section in patients who had been given neoadjuvant chemotherapy; and (2) the accurate localization of the tumour site in patients with cCR or cPR with minimal disease. Kuerer *et al* recommend radiological placement of metallic markers pre-chemotherapy especially in women with tumours of less than 2 cm to facilitate adequate resection and pathologic processing [24]. Kurbet *et al* also advocate intra-operative evaluation of the margins [25]. We agree with this and believe it will permit easier and more accurate surgery. Cosmetic results and patient satisfaction in our experience were acceptable, although those who had a lumpectomy had better results than those who underwent a quadrantectomy.

Scholl *et al* and Mauriac *et al* both demonstrated in randomized trials a survival benefit for the neoadjuvant chemotherapy group [26,27]. Other studies, including the NSABP-18 and EORTC 10902 trial, were unable to show any significant difference in overall survival, disease-free survival or loco-regional control. This is supported by most other authors, none of whom were able to demonstrate a survival benefit; however all the trials were able to demonstrate objective clinical response and decrease in the rate of mastectomy. Moreover, this was not associated with an increase in local recurrence. In the EORTC 10902 trial, investigators compared preoperative versus post-operative chemotherapy in breast cancer, where they found that the breast-conserving therapy rate was higher in the preoperative chemotherapy group in comparison with the post-operative chemotherapy group. This finding, together with the equal loco-regional control rate in both groups, advocates the advantageous role of primary chemotherapy in breast-conserving management [9,14,23].

In large trials, although there was no overall survival difference between neoadjuvant and adjuvant chemotherapy arms, a difference in overall survival, disease-free survival and loco-regional control was observed in several subsets of patients. Survival was more significantly improved in the subset of patients who achieved pCR than in the other subsets of patient and than those receiving adjuvant chemotherapy. Likewise, survival was significantly better in patients who had an axillary pCR than in those who did not. Kuerer *et al* went further and stratified patients into three groups. They found the worst survival was for patients with gross axillary disease post-chemotherapy, followed by those with occult micro-metastases, and the best survival was recorded for patients with pCR. These findings are supported by several other authors [28–30]. In our patients, the issues of survival and long-term loco-regional control were not addressed due to the low number of cases and the short follow-up period.

The chemotherapy regimen used in our study was well tolerated and not associated with any major side effects necessitating discontinuation of the treatment. This is in accordance with most other studies that reported the chemotherapy regimens were well tolerated with minimum morbidity [14,18].

In locally advanced and primary inoperable breast cancer, the purpose of preoperative treatment is to enable adequate local treatment. In patients with stage I or II breast cancer who are candidates for breast-conserving therapy irrespective of preoperative chemotherapy, the goal of preoperative chemotherapy is unclear. However, in stage I or II breast cancer patients who are not candidates for BCS, preoperative chemotherapy will definitely increase the proportion who may avoid a mastectomy. Some investigators argue that tumour response to preoperative chemotherapy is an independent predictor of treatment outcome. Therefore, it could be of benefit for breast cancer patients to adjust systemic adjuvant treatment at an early stage if tumour response to preoperative chemotherapy is inadequate. Controversially, preoperative chemotherapy might lead to over-treatment of breast cancer patients. This can be explained by the fact that patients receive systemic treatment regardless of histological staging of the tumour and axillary nodal status.

Moreover, the possibility of studying the effects of chemotherapy on well-established tumour characteristics as well as experimental tumour markers makes chemotherapy in the preoperative setting highly attractive for research purposes [31]. The comparison of core needle biopsies with the same tumour after systemic treatment is a worthwhile reason to continue preoperative chemotherapy trials in early breast cancer.

Unfortunately, not much data concerning quality-of-life issues in relation to preoperative chemotherapy are available in the literature. Quality-of-life studies, however, have been performed to investigate the effects of breast-conserving therapy versus mastectomy and show a less impaired body image for the conservative treatment modality [32]. Considering the fact that preoperative as well as post-operative chemotherapy seem to yield similar results in terms of prognosis, this might be a conclusive factor on the decision of which chemotherapeutic strategy should be chosen. Therefore, the role of preoperative chemotherapy should be studied in future trials that focus on research, equivalence, quality of life, and local control, in addition to better prognosis in patient subsets.

## Conclusion

The use of neoadjuvant chemotherapy in operable breast cancer in this study was associated with tumour and axillary downstaging, which increased the proportion of cases undergoing breast conservation, with acceptable side effects and reasonable cost. The results from our study are in accordance with the most important studies looking at the same question. During the limited follow-up time of this study, no loco-regional recurrences were recorded and one distant treatment failure was recorded. Its impact, if any, on overall or disease-free survival was not addressed in this study. Larger multi-centre randomized studies with a long follow-up are needed to compare the overall and disease-free survival benefit of this treatment modality, especially in different subtypes.

## Figures and Tables

**Figure 1:**
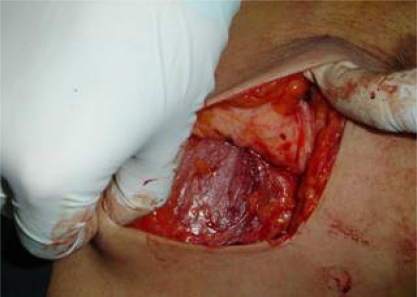
Completion of lumpectomy (note resection of pectoral fascia)

**Figure 2:**
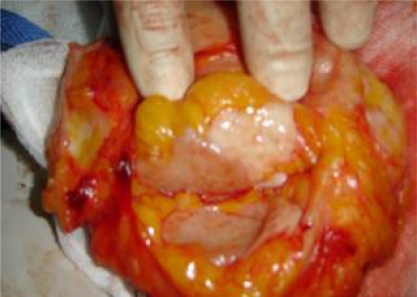
Bisected lumpectomy specimen with grossly adequate margins

**Figure 3:**
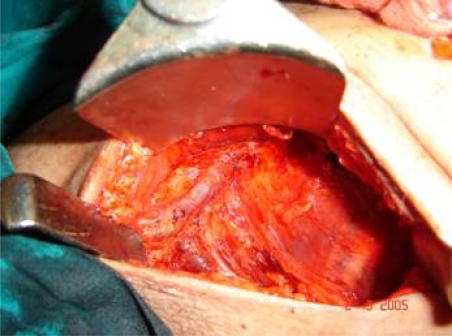
Full axillary dissection, showing vein and nerve to serratus anterior

**Figure 4a:**
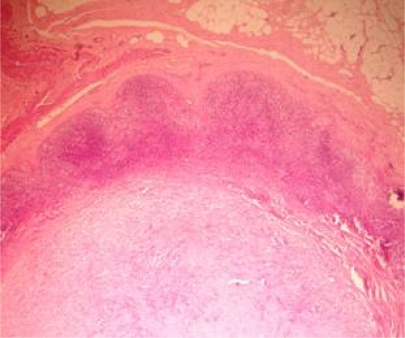
Lymph node, showing partial replacement by evidence of tumour regression (H&E x200)

**Figure 4b:**
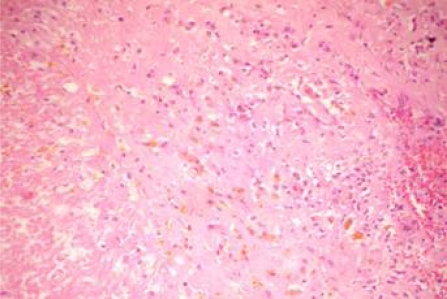
High-power view of Figure 4a, showing from right to left fibrosis, aggregates of foamy histiocytes and necrosis (H&E x400)

**Table 1:**
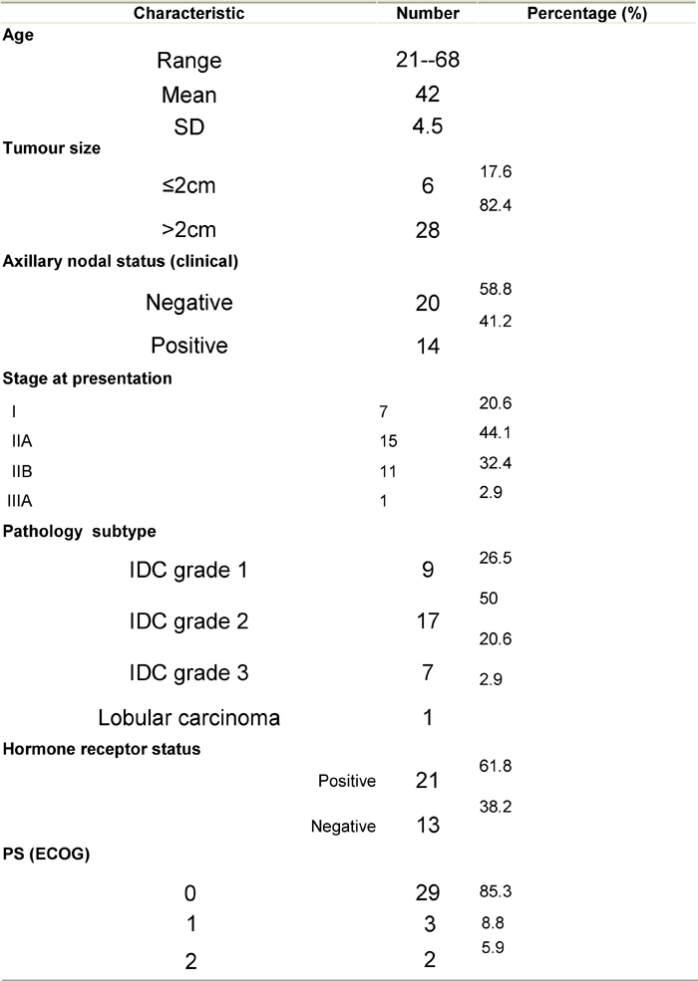
Patient characteristics at presentation

**Table 2:**
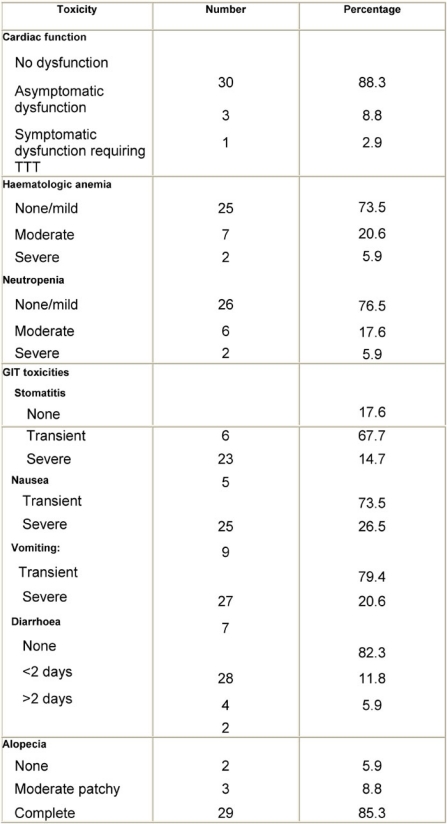
Chemotherapy toxicity profile for the 34 patients of the study

**Table 3:**
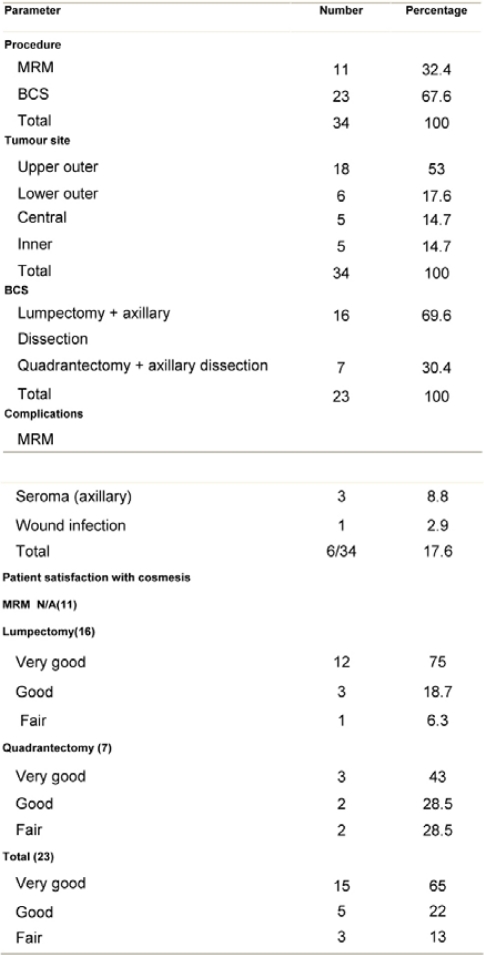
Surgery

**Table 4:**
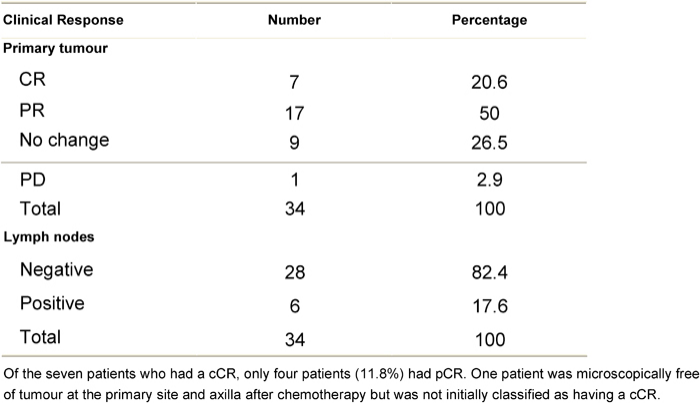
Clinical tumour response after chemotherapy

**Table 5:**
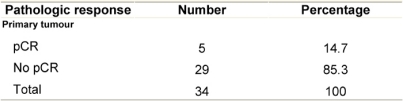
Pathologic tumour response after chemotherapy

**Table 6:**
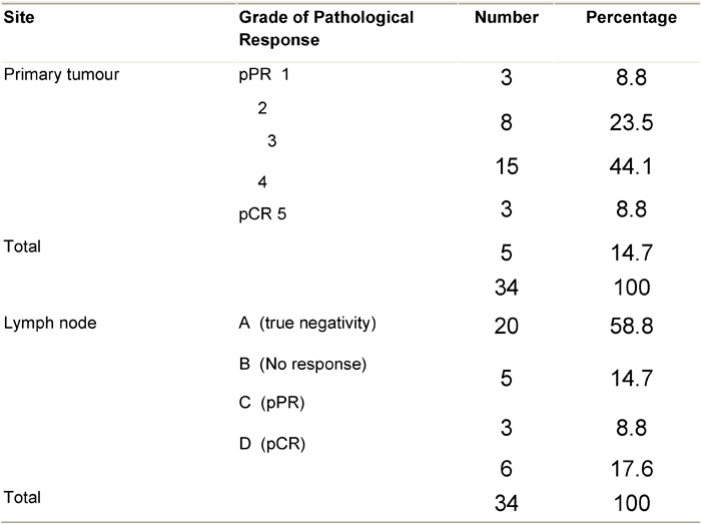
Pathologic tumour response (graded) after chemotherapy
